# Electrochemical Diffusion Study in Poly(Ethylene Glycol) Dimethacrylate-Based Hydrogels

**DOI:** 10.3390/s24113678

**Published:** 2024-06-06

**Authors:** Eva Melnik, Steffen Kurzhals, Giorgio C. Mutinati, Valerio Beni, Rainer Hainberger

**Affiliations:** 1 Molecular Diagnostics, AIT Austrian Institute of Technology GmbH, 1210 Vienna, Austria; steffen.kurzhals@ait.ac.at (S.K.); giorgio.mutinati@ait.ac.at (G.C.M.); rainer.hainberger@ait.ac.at (R.H.); 2 Bioelectronics and Organic Electronics, Smart Hardware, Digital Systems, RISE Research Institutes of Sweden, 60233 Norrköping, Sweden; valerio.beni@ri.se

**Keywords:** hydrogels, electrochemical sensors, diffusivity study, methylene blue, MB-conjugated proteins

## Abstract

Hydrogels are of great importance for functionalizing sensors and microfluidics, and poly(ethylene glycol) dimethacrylate (PEG-DMA) is often used as a viscosifier for printable hydrogel precursor inks. In this study, 1–10 kDa PEG-DMA based hydrogels were characterized by gravimetric and electrochemical methods to investigate the diffusivity of small molecules and proteins. Swelling ratios (*SRs*) of 14.43–9.24, as well as mesh sizes ξ of 3.58–6.91 nm were calculated, and it was found that the *SR* correlates with the molar concentration of PEG-DMA in the ink (*MCI*) (SR = 0.1127 × MCI + 8.3256, R^2^ = 0.9692) and ξ correlates with the molecular weight (*M*_w_) (ξ = 0.3382 × *M*_w_ + 3.638, R^2^ = 0.9451). To investigate the sensing properties, methylene blue (MB) and MB-conjugated proteins were measured on electrochemical sensors with and without hydrogel coating. It was found that on sensors with 10 kDa PEG-DMA hydrogel modification, the DPV peak currents were reduced to 92 % for MB, 73 % for MB-BSA, and 23 % for MB-IgG. To investigate the diffusion properties of MB(-conjugates) in hydrogels with 1–10 kDa PEG-DMA, diffusivity was calculated from the current equation. It was found that diffusivity increases with increasing ξ. Finally, the release of MB-BSA was detected after drying the MB-BSA-containing hydrogel, which is a promising result for the development of hydrogel-based reagent reservoirs for biosensing.

## 1. Introduction

Biosensors enable the rapid and sensitive monitoring of analytes in human body fluids and are thus highly promising in realizing rapid tests and point-of-care devices [1]. Electrochemical sensors are reliable transducers, can be produced cost-effectively using roll-to-roll screen printing, and are suitable for a wide range of analytes in various medical and environmental applications [2,3,4,5]. The assays used for detection usually differ only marginally from the assays used in gold standard methods such as microarrays, lateral flow tests, or enzyme-linked immunosorbent assays [6,7,8,9,10,11]. This is because of the high selectivity and specificity of the established and well-researched assays, which, however, may require sample preparation steps (e.g., target analyte extraction, sample dilution) and target analyte preparation steps (e.g., the addition of chemicals, proteins, enzymes, or antibodies). In this respect, well-designed microfluidic systems, such as those produced by injection molding, micromilling, PDMS punching, or roll-to-roll compatible lithographic processes [12,13], support the automation of sample handling. To make microfluidics operational for various applications in molecular diagnostics, the integration of reagents is a fundamental challenge. Various concepts have been pursued for this purpose, such as blister bags, freeze-dried pellets, functionalized beads, sponges, paper strips, biopolymers, and hydrogels [14,15,16,17,18]. For the large-scale production of microfluidics, automated pick-and-place tools, lamination systems, and spotting and printing technologies have been developed for the assembly process [14].

Among these techniques, spotting methods are particularly suitable because they are also used for the functionalization of biosensor systems [19,20,21,22] and are compatible with roll-to-roll manufacturing processes [13]. Moreover, they can be used for spotting liquid reagents in a microfluidic chamber. In order to increase the shelf life of the reagents, they are commonly embedded in biopolymers or hydrogel networks [23,24,25,26]. In contrast to polymers, hydrogel networks are insoluble and specifically release only the desired substance. This is a decisive advantage, as it prevents a change in viscosity and thus the flow and reaction properties. In addition to their role as reservoirs, hydrogels also play the role of protective and filter layers in biosensing [27,28,29,30], e.g., to prevent nonspecific blood cell binding on the sensor surface [22,30].

In this study, poly(ethylene glycol) di-methacrylate (PEG-DMA)-based hydrogels are investigated in combination with electrochemical sensors. The main focus lies in the diffusion properties of biomolecules through hydrogel films attached to the sensor element in order to use the hydrogels as reagent reservoirs and/or molecular filters. Despite various other methods described in the literature [31,32,33,34], we investigate the diffusion of biomolecules using electrochemical (EC) screen-printed sensors. To better understand the diffusion properties, PEG-DMA-based inkjet printable hydrogel inks with 5.4% (*w*/*w*) 1–10 kDa PEG-DMA are formulated using di(ethylene glycol) vinyl ether (DEGVE) as a monomer and lithium phenyl (2,4,6-trimethylbenzoyl) phosphinate (LAP) as a photoinitiator [22]. 

**Figure 1:**
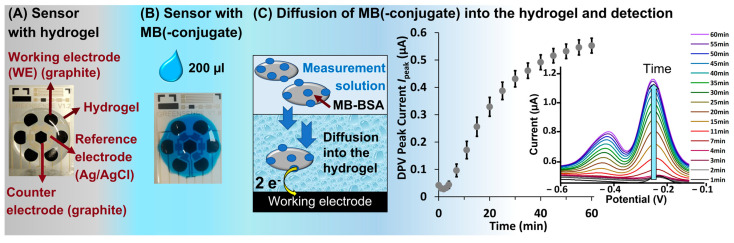
(**A**) Sensors covered with a hydrogel are overlayed with (**B**) MB(-conjugate) solution for (**C**) electrochemical diffusion monitoring with DPV.

Figure 1 depicts the workflow of this study. It starts with the modification of the EC screen-printed sensor with hydrogel (Figure 1A). The characterization of the hydrogels via a gravimetric method enables the calculation of the swelling ratio, the polymer content, the water content, the average molecular weight of polymer chains between two crosslinks (${\bar{M}}_{c}$), and the mesh size (ξ) [33,35]. For the EC measurement, the hydrogel is then overlayed with a methylene blue (MB) or MB-conjugate protein solution (Figure 1B). As proteins, bovine serum albumin (BSA) and mouse IgG are chosen because they are good representatives for bioanalytical assays [14,18]. The measurement is immediately started after applying the MB-(conjugate), and the diffusion is monitored using differential pulse voltammetry (DPV) (Figure 1C). In order to understand the diffusion properties, the following experiments are carried out: (i) measurement of MB(-conjugates) on sensors without hydrogel, (ii) measurement of MB(-conjugates) on sensors coated with a 10 kDa PEDG-DMA hydrogel, (iii) measurement of MB(-conjugates) on sensors coated with different molecular weight (MW) PEG-DMA hydrogels, and (iv) measurement of MB-BSA on sensors coated with wet and dry hydrogels. 

## 2. Materials and Methods

### 2.1. Materials

Mouse IgG, bovine serum albumin (BSA), hexaamine ruthenium (III) chloride [Ru(NH_3_)_6_]Cl_3_, Poly(ethylene glycol) dimethacrylate (PEG-DMA) 10 kDa, 2 kDa, and 1 kDa, di(ethylene glycol) vinyl ether (DEGVE), lithium phenyl (2,4,6-trimethylbenzoyl) phosphinate (LAP), and Dimethyl sulfoxide (DMSO), as well as the chemicals for the physiological phosphate-buffered saline buffer referred to as PBS buffer (10 mmol/L Phosphate, 137 mmol/L NaCl, 2.7 mmol/L KCl; pH 7.3), were purchased from Sigma Aldrich Europe (St. Gallen, Switzerland).

PEG-DMA 5 kDa was purchased from Biopharma PEG Scientific Inc., Watertown, NY, USA. PEG-DMA 3.4 kDa from Alfa Aesar (Haverhill, MA, USA) and the dialysis membrane 3.5 kDa Side-A-Lyser from Thermo Fisher Scientific were purchased from VWR Austria (Vienna, Austria). The methylene-blue N-hydroxy succinimide ester (MB-NHS) for labeling the biomolecules was purchased from Biotium, Fremont, MA, USA. 

### 2.2. Electrochemical Sensors and Measurement Setup

**Figure 2:**
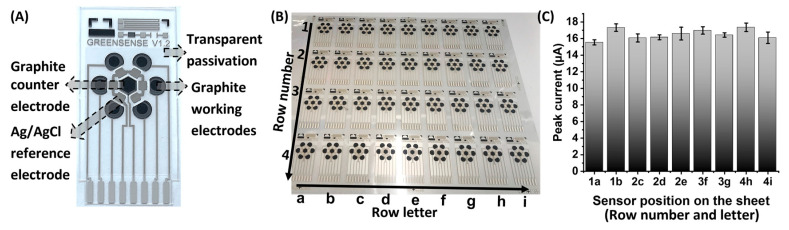
(**A**) Graphite sensor, (**B**) screen-printed sheet, and (**C**) peak currents measured on nine sensors of the screen-printed sheet.

Screen-printed electrochemical (EC) sensor arrays with graphite working electrodes (WE) were produced by RISE Research Institutes of Sweden (Digital Systems, Smart Hardware, Bio- and Organic Electronics, Norrköping, Sweden) according to the AIT design elaborated in the GREENSENSE Project (European Union’s Horizon 2020 research and innovation program under Grant Agreement No. 761000). The screen-printed sensor array consists of six graphite working electrodes (WEs) surrounding a central counter electrode (CE) and a common silver/silver chloride reference electrode (RE) on a PET substrate (Figure 2A). This configuration enabled the measurement of six values simultaneously (WEs = 6). The working electrode area was determined by using a microscope on ten sensors at different positions on the screen-printed sheet (see Section 3.1). For the electrochemical characterization, a 1000 µM hexaamine ruthenium(III) chloride [Ru(NH_3_)_6_]Cl_3_ solution in 50 mmol/L NaCl or MB- and MB-conjugates (see Section 2.6) were used. As the electrochemical measurement method, DPV was chosen with the following parameters: equilibration time *t*_equ_ = 8 s, initial potential *E*_i_ = −0.6 V, final potential *E*_f_ = −0.1 V, step potential *E*_s_ = 0.005 V, interlevel potential *E*_p_ = 0.05 V, step time *t*_p_ = 0.005 s, and scan rate *SR* = 0.5 V/s. A PalmSens MUX8 R2 potentiostat (Figure S1) was used for the DPV measurements. The monitoring measurements of MB(-conjugates) were carried out for up to 160 min (one DPV per minute, or per five minutes), and the DPV peak currents *I*_peak_ were recorded. From the *I*_peak_, the diffusivity (also called diffusion coefficient) *D* of the electrochemical species can be calculated according to Equation (1) [35].

(1) $$\;I_{peak}=\frac{nFAD^{\frac{1}{2}}C}{\pi^{\frac{1}{2}}t_{p}^{\frac{1}{2}}}\left(\frac{1-\sigma }{1+\sigma }\right),\;with\;\sigma =e^{\frac{2FE_{p}}{2RT}}$$

In Equation (1), *n* is the number of electrons (2 for methylene blue, and 2 multiplied by the degree of labeling for BSA or IgG, which are 5.4 and 3.8, respectively (see Section 2.6)), *F* is the Faraday constant (96,485 C × mol^−1^), *A* is the working electrode area (see Section 3.1), *C* is the concentration of the analyte, *R* is the gas constant, and *T* the temperature in Kelvin (293.15 K = 20 °C). 

### 2.3. Hydrogel Preparation

The hydrogel inks were fabricated by mixing 30 µL of 166 mg/mL PEG-DMA (1–10 kDa) in water with 60 µL di(ethylene glycol) vinyl ether (DEGVE) and 2 µL of a lithium phenyl (2,4,6-trimethylbenzoyl) phosphinate (LAP) solution (10 mg LPA per 100 µL in a 1:1 mixture of ethanol and ultrapure water). The PEG-DMA weight percent was kept constant at 5.4 % (*w*/*w*) in each ink by decreasing the molar concentration (µmol/L) with respect to the increase in molecular weight of PEG-DMA (see Section 3.2). This strategy was chosen to fulfill the following requirements: (a) the inks should have a viscosity of <3.0 cP (to be applicable later in a printing process) and (b) the hydrogels should have good solidity after crosslinking. Preliminary tests showed that the inks prepared with 5.4% (*w*/*w*) of 1, 2, 3.4, and 10 kDa PEG-DMA composition fulfilled these criteria. No hydrogel with good solidity could be prepared with 5.4% (*w*/*w*) of 5 kDa PEG-DMA. As a consequence, only the 1, 2, 3.4, and 10 kDa hydrogel could be investigated in this study. 

For the hydrogel preparation on the EC-sensor, 80 µL of the inks were applied and UV-crosslinked at a wavelength of 365 nm (1 J/cm^2^) using the UVP crosslinker CL-3000 (Analytik Jena US, Jena, Germany). The crosslinking step forms acrylate radicals that are highly reactive and can bind to untreated organic surfaces. This process is so strong that even screen-printed graphite-based sensor surfaces can be modified with hydrogels without any pre-treatment. This process led to an approximately 2 mm thick wet hydrogel layer on the sensor surface, which was washed two times with PBS buffer (1 × 10 min, 1 × 15 min) (Figure 1A). The hydrogel layers were tested in wet (Figure 1A) and dry states. 

### 2.4. Gravimetric Hydrogel Characterization

The gravimetric analysis of hydrogels allows for calculating parameters that characterize the mesh structure of the hydrogel [36]. Therefore, hydrogel structures were fabricated, as described in Section 2.3, and gravimetric analyses were performed using a sensitive scale. For each PEG-DMA ink variation, a set of three sensors was prepared with hydrogel coatings, and the weights and volumes were determined. The variables used for this analysis are also listed in Table S1. 

For a better understanding of water uptake and, therefore, of the hydrophilic properties of the hydrogel, the swelling ratio (*SR*) and water content of the hydrogel was calculated. The weight of the screen-printed sensor (*W*_sens_), the weight of the washed hydrogel on the sensor (*W*_wash_), and the weight of the dried hydrogel on the sensor after drying for 24 h at room temperature (*W*_dry_) were determined. The weight of the swollen hydrogel (*W*_sh_) was calculated by *W*_sh_ = *W*_wash_ − *W*_sens_ and the weight of the polymer (*W*_p_) by *W*_p_ = *W*_dry_ − *W*_sens_. The SR was finally calculated according to the equation *SR* = *W*_sh_/*W*_p_. The weight of the water in the hydrogel (*W*_w_) was determined according to the equation *W*_w_ = *W*_wash_ − *W*_dry_. 

Another interesting parameter is the number-average molecular weight of the polymer chains between crosslinks (${\bar{M}}_{c}$). Using this parameter in combination with other variables, the mesh size ξ of the hydrogel can be calculated. As ${\bar{M}}_{c}$ and ξ allow for different hydrogels to be compared with each other, they are the primary objective of this calculation. The ${\bar{M}}_{c}$ can be calculated using Equation (2) [37].

(2) $$\frac{1}{{\bar{M}}_{c}}=\frac{2}{{\bar{M}}_{n}}-\frac{\tilde{v}/V_{1}[\ln\left(1-v_{2,s}\right)+v_{2,s}+\chi v_{2,s}^{2}]}{v_{2,r}[{\left(\frac{v_{2,s}}{v_{2,r}}\right)}^{\frac{1}{3}}-\frac{v_{2,s}}{v_{2,r}}]}$$

In Equation (2), ${\bar{M}}_{n}$ is the number-average molecular weight of PEG-DMA, $\tilde{v}$ is the specific volume of the polymer ($\tilde{v}$ = 1/ρ_p_ with ρ_p_ being the density of the polymer of 1.1 kg × L^−1^), *V*_1_ is the molar volume of the solvent (18 × 10^−3^ L × mol^−1^ for water), χ is the Flory–Huggin’s polymer–solvent interaction parameter (0.495 for PEG-water system) [37], *v*_2,*s*_ is the volume fraction of the swollen gel, and *v_s_*_,*r*_ is the volume fraction of the relaxed gel (gel after crosslinking). The parameter *v*_2,*s*_ is given by *v*_2,*s*_ = V_p_/V_s_, where *V*_p_ is the volume of the polymer and *V*_s_ the volume of the gel. The parameter v_2,r_ is given by *v*_2,*s*_ = *V*_p_/*V*_r_, where *V*_r_ is the relaxed volume of the gel. From ${\bar{M}}_{c}$, the mesh size ξ can be calculated using Equation (3) [38]

(3) $$\xi =v_{2,s}^{-1/3}\;*\;C_{n}^{\frac{1}{2}}{\left(\frac{2{\bar{M}}_{c}}{M_{r}}\right)}^{\frac{1}{2}}\;*\;l$$

In Equation (3), *C_n_* is the rigidity factor of the polymer (4 for PEG) [39], *M_r_* is the molecular weight of repeating units (44 × 10^−3^ kg/mol for PEG), and l is the carbon–carbon bond length (0.154 nm). 

### 2.5. Hydrogel Characterization with Scanning Electron Microscopy

For SEM imaging analyses, 10 kDa PEG-DMA hydrogels were processed on a screen-printed Ag layer to provide an electrical contact to the sample. Next, lyophilization was performed in a Christ Alpha 2–4 LCSplus freeze dryer. The freeze dryer was set, and the pre-cooling was switched on. The settings are listed in Table 1. During the warm-up of the system, two of the sensors were shock-frozen in liquid nitrogen. Subsequently, they were transferred into the freeze drier, and the main drying process was performed overnight. After lyophilization, the sensors were stored under vacuum until further use. The sputtering of a gold/palladium mixture was deliberately omitted, as otherwise, the fine structure of the hydrogel would no longer have been visible (Figure S2).

A ZEISS Gemini SEM was used to image the hydrogel layers after drying in vacuum (in the desiccator) and after lyophilization. SEM imaging was performed with a SE2 secondary electron detector at an electron high tension (EHT) voltage of 5 kV.

### 2.6. Conjugation of Biomolecules with Methylene Blue

To obtain MB-BSA (or MB-IgG) conjugates, 300 µL of a 5 mg/mL BSA or IgG solution in physiological PBS and 121 µL for BSA (53 µL for IgG) of a 5 mg/mL solution of MB-NHS in DMSO were mixed and incubated for 1.5 h at room temperature. The MB-NHS/protein solutions were then dialyzed in a 3.5 kDa Side-A-Lyser against physiological PBS buffer at 4 °C for 12 h. Then, the liquids were transferred to a 1 mL volumetric flask and filled up to 1 mL using physiological PBS buffer. By using UV-VIS absorption measurements, a degree of labeling (DOL) of 5.4 could be detected for MB-BSA and 3.8 for MB-IgG.

## 3. Results

### 3.1. Sensor Characterization

The area of the working electrode was determined on ten sensors by a microscope image analysis, which comprised sixty working electrodes. The measured areas showed good homogeneity of 2.13 ± 0.05 mm^2^. Functionality and reproducibility were tested with a 1000 µmol/L hexaamine ruthenium(III) chloride [Ru(NH_3_)_6_]Cl_3_ solution in 50 mmol/L NaCl by performing DPV (Figure 2C) on nine sensors (six working electrodes each) taken from different positions on the screen-printed sheet (Figure 2B). The average peak current amounted to 16.44 ± 0.61 µA.

### 3.2. Gravimetric Hydrogel Characterization

Hydrogels with different PEG-DMA molecular weights were prepared as described in Section 2.3. Gravimetric analyses were performed according to Section 2.4. The swelling ratio, the number-average molecular weight of the polymer chain between the crosslinks, ${\bar{M}}_{c}$ (g/mol), and the mesh size ξ (nm) were calculated for each PEG-DMA hydrogel. Table 2 summarizes the results. (A more detailed list is given in Table S2).

It was found that the higher the molecular weight of PEG-DMA, the lower the degree of swelling (*SR*). This may seem surprising at first glance. However, as shown in Table 2, the molar concentration in the ink (*MCI*) decreases with the increasing molecular weight of PEG-DMA. Therefore, there are more dimethacrylate crosslink points available in the 1 kDa PEG-DMA ink than in the 10 kDa PEG-DMA ink, which affects the swelling ratio. Linear fitting revealed that the two quantities correlate via the linear equation *SR* = 0.1127 × *MCI* + 8.3256 with an R^2^ = 0.9692 (Figure S3A), which proves the plausibility of the assumption. The swelling ratio does not correlate linearly with the molecular weight of PEG-DMA (*M*_w_) (Figure S3B). However, a clear linear correlation between the mesh size and the molecular weight of PEG-DMA was found with ξ = 0.3382 × *M*_w_ + 3.638, with an R^2^ = 0.9451 (Figure S3D).

### 3.3. Hydrogel Characterization with Scanning Electron Microscopy

**Figure 3:**
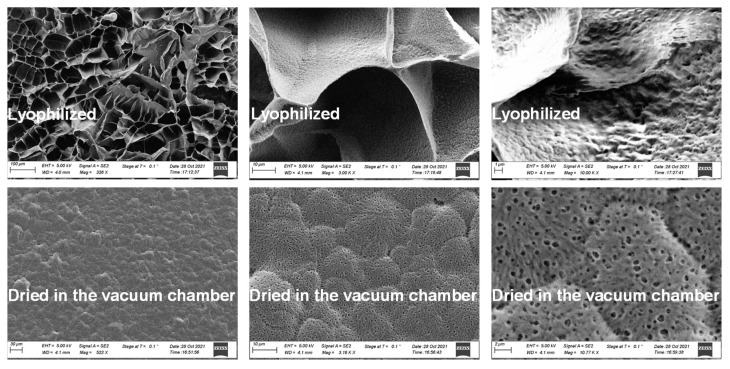
SEM images of vacuum-dried and lyophilized hydrogel structures.

To better understand the 3D structure of the PEG-DMA hydrogel, SEM images of the 10 kDa PEG-DMA hydrogel were taken after lyophilization and after drying in the vacuum chamber, which reflects the wet and dry structure of the hydrogel. At magnifications <600, the lyophilized hydrogel shows an extended network structure that appears to be interrupted by polymer walls, in contrast to the vacuum-dried hydrogel. At magnifications of around 3000, only the vacuum-dried hydrogel shows a clear pore structure with a pore diameter of about 1 µm. With a magnification of 10,000, a nano-pore structure becomes visible on the polymer wall of the lyophilized hydrogel, although this is very difficult to resolve with the SEM (see Figure 3).

### 3.4. MB-Conjugate Measurement without Hydrogel

**Figure 4:**
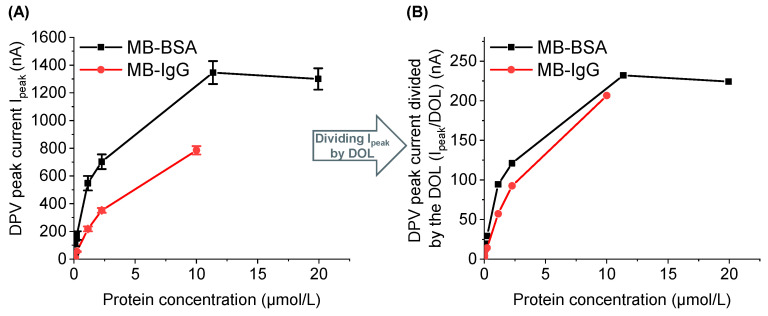
(**A**) Concentration-dependent measurement of MB-BSA and MB-IgG on unmodified sensors. (**B**) Normalization of the DPV peak current I_peak_ with the DOL of 5.8 for MB-BSA and 3.8 for MB-IgG.

The MB-conjugation leads to a redox-labeling of the biomolecules. In the first experiment, different concentrations of MB(-conjugates) in physiological PBS buffer were measured on sensors without a hydrogel layer. It was found that when the DPV *I*_peak_ values (at 0 min) for MB-BSA and MB-IgG conjugates are normalized by dividing them by the degree of labeling (DOL), comparable results are obtained for both conjugates. This indicates that conjugated MB molecules equally contribute to the current output (Figure 4).

### 3.5. Response of MB-Conjugates of Sensors with and without Hydrogel

**Figure 5:**
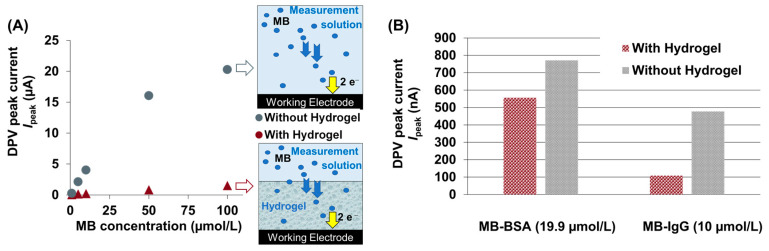
(**A**) Detection of MB with and without a hydrogel coating. (**B**) Detection of MB-BSA (19.9 µmol/L) and MB-IgG (10 µmol/L) with and without a hydrogel coating.

The unmodified sensors and the sensors modified with a 10 kDa PEDG-DMA hydrogel were measured with MB(-conjugates) for 60 min to allow the signals to equilibrate (see Figure S4). A DPV measurement was performed every minute for the first five minutes of the test and every five minutes thereafter. The DPV *I*_peak_ current values after 60 min were plotted versus the concentration of MB. Figure 5A shows the concentration-dependent measurement results of methylene blue (MB) on EC sensors with and without a hydrogel coating. The DPV Ipeak values were found to have decreased to 8% for MB (Figure 5A), to 73% for MB-BSA (Figure 5B) and to 23% for MB-IgG (Figure 5B) compared to the results of the unmodified sensor. Furthermore, the DOL normalization of the DPV *I*_peak_ currents (see Section 3.4) obtained from the sensors with the hydrogel coating did not lead to comparable results for MB-BSA and MB-IgG. This indicates different transport properties of BSA and IgG in the hydrogel. However, the results demonstrate a good migration property of MB(-conjugates) through the ~2 mm thick wet hydrogel.

### 3.6. Hydrgel Characteriazion with Different PEG-DMA Molecular Weights

Hydrogels with different MW PEG-DMAs (1, 2, 3.4, and 10 kDa) were compared regarding the diffusion properties for the MB(-conjugates). For this purpose, 50 µmol/L MB, 22.7 µmol/L MB-BSA, and 10 µmol/L MB-IgG were measured on the sensors modified with the different hydrogel layers.

The hydrogel layers were used directly after washing with PBS, without an intermediate drying step. The measurements were started after the application of the MB(-conjugate) solutions and performed for 60 min. 

The diffusivity of the MB(-conjugates) was calculated using Equation (1), and the peak currents after 60 min on the sensor with or without hydrogel were used. 

**Figure 6:**
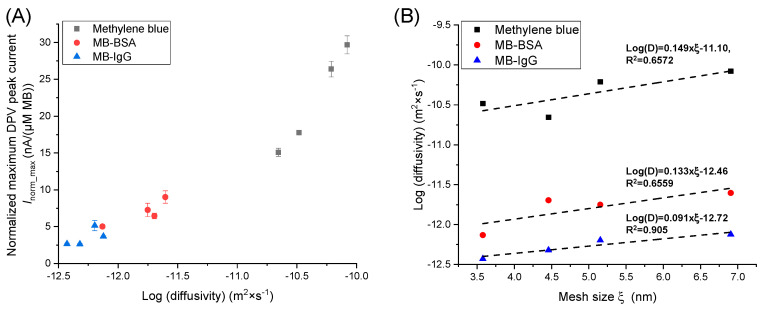
Plots of (**A**) I_norm_max_ versus diffusivity and (**B**) diffusivity versus mesh size ξ.

Table 3 shows the results of the calculated diffusivity. Measurements without hydrogel result in electrochemical diffusivity values for MB, MB-BSA, and MB-IgG with a deviation from the values reported in the literature of 6.74 × 10^−10^ [40], 5.9 × 10^−11^ [32,33,41], and 4.0 × 10^−11^ m^2^ × s^−1^ [33,41], respectively. This deviation might be explained by the fact that the literature values were calculated values using the hydrodynamic radius of the molecules and did not consider the migration properties under the given conditions (temperature, buffer solution composition) or alternated migration properties of the loaded MB(-conjugates) in electrochemical fields. Furthermore, the diffusivity of the MB-conjugates is significantly reduced in the case of hydrogel modification on the sensor (see Table 3). For a better comparison of the current values, the peak currents were normalized with respect to the MB concentration in µM (for MB, *I*_norm_= *I*_peak_/(MB concentration in µM), for MB-BSA and MB-IgG, *I*_norm_= *I*_peak_/(DOL × protein concentration in µM)) (Figure S5). To compare the maximum current and the equilibrium times, a Langmuir fit was performed using the equation *I*_norm_(*t*) = *I*_norm_max_ × *t*/(*k*_t_ + *t*), where current I_peak_max_ is the maximum equilibration current, *t* is the measurement time of each data point, and *k*_t_ is the equilibrium rate constant (Figure S5, Table S3). The highest *I_norm_max_* was found for MB, followed by MB-BSA and MG-IgG (see Table 3). Figure 6A shows a plot of *I*_norm_max_ versus diffusivity and Figure 6B shows diffusivity versus mesh size ξ, where a clear correlation can be observed.

### 3.7. MB-BSA Diffusion into and out of the Wet Hydrogel and of the Dry Hydrogel

For the fabrication of hydrogel reservoirs, the hydrogel structure must be filled with a reagent. After the drying procedure, this reagent should be able to migrate out of the hydrogel into the supernatant solution. To test this ability, 10 kDa PEG-DMA hydrogels were fabricated on EC sensors, and the MB-BSA diffusion into and out of a wet hydrogel and a dry hydrogel was investigated. 

**Figure 7:**
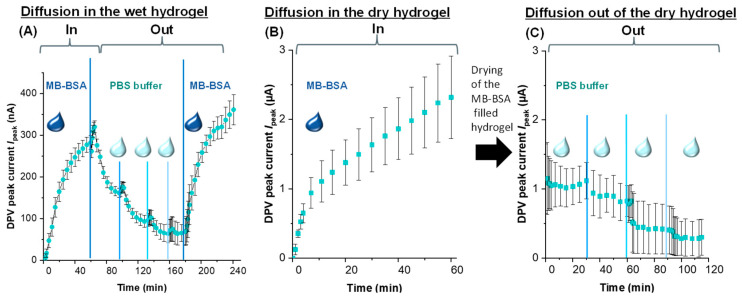
Diffusion study of MB-BSA in the wet (**A**), in the dry hydrogels (**B**), and out of the dry hydrogel (**C**).

Figure 7A shows the EC measurement in the wet hydrogel, where, in the first 60 min, MB-BSA migrated from the top solution (22.7 µmol/L MB-BSA, 200 µL) into the hydrogel until it was evenly distributed in the gel (noticeable by the fact that all six WE showed a stable current) and *I*_peak_ reached the value of 282.96 ± 16.80 nA. Subsequently, the MB-BSA solution was replaced with PBS buffer (200 µL). From this point onwards, the current decreased again as MB-BSA migrated out of the hydrogel, and thus, the redox-active MB moved away from the sensor surface. At minute 90, a new equilibrium was reached with an average *I_peak_* of 165.45 ± 11.25 nA, and the top solution was exchanged again with fresh PBS buffer (200 µL). After 130 min, an equilibrium with 93.12 ± 14.46 nA was reached. After changing the buffer again, the current only dropped to 63.93 ± 22.35 nA at minute 158. A further buffer change did not lead to a further significant drop in current (at 175 min, 65.55 ± 20.34 nA). If this current value is calculated as a percentage of the current after diffusion of MB-BSA in the wet hydrogel, it can be said that around 22 % of the MB-BSA remains in the hydrogel and cannot be washed out any further. Subsequently, the PBS buffer was replaced with an MB-BSA solution, and again, the migration of MB-BSA into the hydrogel could be monitored until minute 241 (*I*_peak_ 361.24 ± 36.48 nA), which proves the reversibility of the diffusion process. 

For the fabrication of hydrogel reservoirs, the prior drying of the hydrogel is important to avoid a dilution of the reagent with the water in the gel. Because of this fact, the hydrogel was dried in vacuum and subsequently, the migration of MB-BSA into the dry hydrogel layer was monitored by EC measurements. Figure 7B depicts the measurement. After the application of the MB-BSA solution (22.7 µmol/L, 200 µL), the signal increased and the hydrogel swelled. After 60 min, the current was 2.32 ± 0.60 µA (n = 6), with a quite high standard deviation, and thus, in a similar µA range as the current without the hydrogel (1.30 ± 0.08 µA for 19.9 µmol/L, see Figure 4A). After the saturation of the hydrogel with MB-BSA, the top solution was removed, and the hydrogel was dried. Drying leads to the inclusion of the reagents in the dry hydrogel polymer structure. The inclusion of proteins in the dense polymeric network of the dry hydrogel can also stabilize their 3D structure [42,43,44] After the drying step, the hydrogel network was overlayed with PBS buffer (200 µL). Figure 7C shows the plot of the current. At minute 30, an equilibrium was reached with an average *I*_peak_ of 1.07 ± 0.26 µA, and the top solution was again exchanged with fresh PBS buffer (200 µL). The reduction in the *I*_peak_ current can be explained by the dilution effect of MB-BSA. The hydrogel can only take up 128.5 µL of the MB-BSA solution (Table S2, 10 kDa *W*_w_), but it becomes diluted with 200 µL PBS buffer in the hydrogel. Then, 60 min after the PBS buffer application, an equilibrium with 0.82 ± 0.23 µA was reached. After changing the buffer again, the current only dropped to 0.40 ± 0.35 µA by minute 90. Another buffer change further reduced the current to 0.28 ± 0.26 µA, which corresponds to 12% of the MB-BSA current after filling and 26% of the current after the first addition of PBS buffer. The latter indicates a reduced mobility of MB-BSA in the dried hydrogel network.

## 4. Discussion

The diffusion properties of biomolecules are an important parameter and must be considered in the production of hydrogels for biosensor applications. In our study, we approached this topic by preparing and characterizing different printable PEG-DMA-based hydrogels. Gravimetric and electrochemical characterization methods were used and finally brought into relation. The gravimetric method showed that the swelling ratio (*SR*) can be adjusted primarily via the molar concentration of the PEG-DMA compound in the hydrogel ink. This is crucial and must be considered if either excessive swelling should be prevented in a microfluidic channel or excessive swelling of the hydrogels is desired, e.g., if hydrogels are used as valves in a microfluidic channel.

The calculated number-average molecular weight of the polymer chain between the crosslinks (${\bar{M}}_{c}$) and mesh sizes ξ are in good agreement with values from the literature [39], where a PEG-DMA mixture was used for the preparation of hydrogels. 

Therefore, the addition of DEGVE to hydrogel inks only has the purpose of ensuring good printability and minimally influences the formation of the network structure. 

The number-average molecular weight of the polymer chain ${\bar{M}}_{c}$ and the mesh size ξ provide information about the strength of the hydrogel and enable the estimation of the diffusion properties of the hydrogel, as demonstrated further below. Several experiments were systematically conducted to investigate the diffusion properties. First, MB(-conjugates) were measured on sensors without the hydrogel. It was found that the degree of labeling (DOL) influences the level of the measured current, and the normalization of the currents enables the direct comparison of MB-conjugates with different DOLs. 

First, this result is decisive because it demonstrates the accuracy of the method of labeling the biomolecules. Moreover, this result also shows that the methylene blue molecules bound to the biomolecules contribute equally to the resulting DPV peak current. This is particularly important for the calculation of the diffusivity from the electrochemical DPV equation (Equation (1)) for the number of electrons per mole of MB(-conjugate) (*n*), as we will discuss further below.

Second, the sensors coated with a 10 kDa PEG-DMA-based hydrogel showed that hydrogel coatings on sensors lead to a reduction in DPV peak currents to 8% for MB, 73% for MB-BSA, and 23% for MB-IgG with respect to the measurements without the hydrogel. This is probably because fewer molecules reach the electrode surface in the presence of a hydrogel coating, leading to limited diffusion. As the sensing kinetics depends significantly on the thickness of the hydrogel layer, the thickness of the hydrogel layer must also be considered when producing filter layers over sensors to avoid a limitation in the sensitivity of the sensor. 

Third, sensors coated with PEG-DMA hydrogels of different molecular weights (*M*_w_) show that a higher *M*_w_ leads to higher equilibrium currents and faster diffusion. As the mesh sizes do not change following the diffusion of MB-BSA and MB-IgG into the hydrogel network, the calculated values from the independent gravimetric method can be correlated with the diffusivity of these molecules, again demonstrating the plausibility of the chosen methods. It was found that the diffusivity increases for MB-BSA, MB-IG, and MB with increasing mesh sizes. This can be explained by increased molecule mobility due to a reduced interaction between the molecules and the polymer chains of the hydrogel network.

Fourth, wet and dry hydrogels were used to study the diffusion of MB-BSA. It was found that up to 88 % of the MB-BSA introduced into dried hydrogels could be released from the hydrogel. Therefore, the hydrogel inks investigated in this study have a good potential to be used as a reagent reservoir. 

In future studies, the use and long-term stability of hydrogels as reagent reservoirs on sensors and in microfluidics will be investigated. Spotting and dispensing methods, as already demonstrated for the measurement of lactate on microneedles [22], will be investigated for printing in microfluidics.

## Figures and Tables

**Table 1:** Settings for the lyophilization.

	Freeze	Warm-Up	Main Drying	Post-Drying
Time (min)	90	30	Setting: ∞	20
Temperature (°C)	−40	−50	−50	25
Vacuum (mbar)			0.015	0.015
Pressure (mbar)			off	off

**Table 2:** Values of the swelling ratio, the number-average molecular weight of the polymer chain between the crosslinks ${\bar{M}}_{c}$ (g/mol), and mesh size ξ (nm).

Molecular Weight of PEG-DMA, *M*_w_ (kDa)	Molar Concentration in the Ink, MCI (µmol/L)	Swelling Ratio, *SR* = *W*_sh_/*W_p_*	Number-Average Molecular Weight of Polymer Chain between CrossLinks, ${\bar{M}}_{c}$ (g/mol)	Mesh Size, ξ (nm)
1	54.1	14.43	407.37	3.58
2	27.1	11.66	630.38	4.46
3.4	15.9	9.53	900.61	5.15
10	5.4	9.24	1238.22	6.91

**Table 3:** Results of the comparison of different PEG-DMA hydrogels for the sensing of MB(-conjugates).

PEG-DMA (kDa)	Methylene Blue		MB-BSA		MB-IgG	
	Diffusivity (m^2^ × s^−1^)	*I*_norm_max_ ± sd, (nA/(µM MB)	Diffusivity (m^2^ × s^−1^)	*I*_norm_max_ ± sd, (nA/(µM MB)	Diffusivity (m^2^ × s^−1^)	*I*_norm_max_ ± sd, (nA/(µM MB)
**no** **hydrogel**	1.6 × 10^−8^		4.8 × 10^−12^		1.5 × 10^−11^	
**1**	3.3 × 10^−11^	17.8 ± 0.2	7.4 × 10^−13^	5.0 ± 0.2	3.7 × 10^−12^	2.6 ± 0.1
**2**	2.2 × 10^−11^	15.1 ± 0.6	2.0 × 10^−12^	6.5 ± 0.4	4.8 × 10^−13^	2.6 ± 0.1
**3.4**	6.1 × 10^−11^	26.4 ± 1.1	1.8 × 10^−12^	7.2 ± 0.9	6.4 × 10^−13^	5.1 ± 0.7
**10**	8.3 × 10^−11^	29.7 ± 1.2	2.5 × 10^−12^	9.0 ± 0.8	7.5 × 10^−12^	3.7 ± 0.1

## Data Availability

The data are contained within this article and Supplementary Materials.

## Supplementary Materials

The following supporting information can be downloaded at: https://www.mdpi.com/article/10.3390/s24113678/s1, Figure S1: Measurement setup: (1) PalmSens multiplexer MUX8-R2 (PSTrace software 5.7), (2) connector, (3) sensor, and (4) computer; Table S1: Variables of the gravimetric analysis; Table S2: Determined values from gravimetric hydrogel characterization; Figure S2: SEM images of vacuum-dried and lyophilized hydrogel structures; Figure S3: Correlation between (A) the swelling ratio and the molar concentration in the ink, (B) the swelling ratio and the molecular weight of PEG-DMA (kDa), (C) mesh size and the molar concentration in the ink, and (D) mesh size and the molecular weight of PEG-DMA (kDa); Figure S4: DPV measurement of electrochemical sensors of MB-BSA and MB-IgG (A) without and (B) with the hydrogel (10 kDa PEG-DMA as crosslinker) over 60 min. During the first five minutes, a DPV measurement was performed every minute and afterward, every five minutes; Figure S5: Normalized DPV peak currents (nA/µM MB) of MB, MB-BSA, and MB-IgG measurements on 1, 2, 3.4, and 10 kDa PEG-DMA hydrogel-modified sensors over 60 min. Curves were fitted with a Langmuir fitting function; Table S3: Values of the equilibrium rate constant *k_t_*.

## References

[1] Kim E.R., Joe C., Mitchell R.J., Gu M.B. (2023). Biosensors for healthcare: Current and future perspectives. Trends Biotechnol..

[2] Gonzalez-Macia L., Morrin A., Smyth M.R., Killard A.J. (2010). Advanced printing and deposition methodologies for the fabrication of biosensors and biodevices. Analyst.

[3] Ronkainen N.J., Halsall H.B., Heineman W.R. (2010). Electrochemical biosensors. Chem. Soc. Rev..

[4] da Silva E.T.S.G., Souto D.E.P., Barragan J.T.C., Giarola J.D.F., De Moraes A.C.M., Kubota L.T. (2017). Electrochemical Biosensors in Point-of-Care Devices: Recent Advances and Future Trends. ChemElectroChem.

[5] Couto R.A.S., Lima J.L.F.C., Quinaz M.B. (2016). Recent developments, characteristics and potential applications of screen-printed electrodes in pharmaceutical and biological analysis. Talanta.

[6] Schrattenecker J.D., Heer R., Hainberger R., Fafilek G. (2017). Impedimetric IgG-Biosensor with In-Situ Generation of the Redox-Probe. Proceedings.

[7] Schrattenecker J.D., Heer R., Melnik E., Maier T., Fafilek G., Hainberger R. (2018). Hexaammineruthenium (II)/(III) as alternative redox-probe to Hexacyanoferrat (II)/(III) for stable impedimetric biosensing with gold electrodes. Biosens. Bioelectron..

[8] Nimse S.B., Sonawane M.D., Song K.-S., Kim T. (2016). Biomarker detection technologies and future directions. Analyst.

[9] Ricci F., Adornetto G., Palleschi G. (2012). A review of experimental aspects of electrochemical immunosensors. Electrochim. Acta.

[10] Thoeny V., Melnik E., Asadi M., Mehrabi P., Schalkhammer T., Pulverer W., Maier T., Mutinati G.C., Lieberzeit P., Hainberger R. (2022). Detection of breast cancer-related point-mutations using screen-printed and gold-plated electrochemical sensor arrays suitable for point-of-care applications. Talanta Open.

[11] Thoeny V., Melnik E., Huetter M., Asadi M., Mehrabi P., Schalkhammer T., Pulverer W., Maier T., Mutinati G.C., Lieberzeit P. (2023). Recombinase polymerase amplification in combination with electrochemical readout for sensitive and specific detection of PIK3CA point mutations. Anal. Chim. Acta.

[12] Faustino V., Catarino S.O., Lima R., Minas G. (2016). Biomedical microfluidic devices by using low-cost fabrication techniques: A review. J. Biomech..

[13] Toren P., Smolka M., Haase A., Palfinger U., Nees D., Ruttloff S., Kuna L., Schaude C., Jauk S., Rumpler M. (2020). High-throughput roll-to-roll production of polymer biochips for multiplexed DNA detection in point-of-care diagnostics. Lab Chip.

[14] Deng J., Jiang X. (2019). Advances in Reagents Storage and Release in Self-Contained Point-of-Care Devices. Adv. Mater. Technol..

[15] Smith S., Sewart R., Becker H., Roux P., Land K. (2016). Blister pouches for effective reagent storage on microfluidic chips for blood cell counting. Microfluid. Nanofluid.

[16] Xu J., Wang J., Su X., Qiu G., Zhong Q., Li T., Zhang D., Zhang S., He S., Ge S. (2021). Transferable, easy-to-use and room-temperature-storable PCR mixes for microfluidic molecular diagnostics. Talanta.

[17] Zirath H., Schnetz G., Glatz A., Spittler A., Redl H., Peham J.R. (2017). Bedside Immune Monitoring: An Automated Immunoassay Platform for Quantification of Blood Biomarkers in Patient Serum within 20 Minutes. Anal. Chem..

[18] Zhao Z., Al-Ameen M.A., Duan K., Ghosh G., Lo J.F. (2015). On-chip porous microgel generation for microfluidic enhanced VEGF detection. Biosens. Bioelectron..

[19] Kirk J.T., Fridley G.E., Chamberlain J.W., Christensen E.D., Hochberg M., Ratner D.M. (2011). Multiplexed inkjet functionalization of silicon photonic biosensors. Lab Chip.

[20] Abe K., Hashimoto Y., Yatsushiro S., Yamamura S., Bando M., Hiroshima Y., Kido J., Tanaka M., Shinohara Y., Ooie T. (2013). Simultaneous Immunoassay Analysis of Plasma IL-6 and TNF-α on a Microchip. PLoS ONE.

[21] Melnik E., Muellner P., Mutinati G.C., Koppitsch G., Schrank F., Hainberger R., Laemmerhofer M. (2016). Local functionalization of CMOS-compatible Si 3 N 4 Mach-Zehnder interferometers with printable functional polymers. Sens. Actuators B Chem..

[22] Kurzhals S., Melnik E., Plata P., Cihan E., Herzog P., Felice A., Bocchino A., O’Mahony C., Mutinati G.C., Hainberger R. (2023). Detection of lactate via amperometric sensors modified with direct electron transfer enzyme containing PEDOT:PSS and hydrogel inks. IEEE Sens. Lett..

[23] Lee J., Ko J.H., Lin E.-W., Wallace P., Ruch F., Maynard H.D. (2015). Trehalose hydrogels for stabilization of enzymes to heat. Polym. Chem..

[24] Panescu P.H., Ko J.H., Maynard H.D. (2019). Scalable Trehalose-Functionalized Hydrogel Synthesis for High-Temperature Protection of Enzymes. Macromol. Mater. Eng..

[25] Mancini R.J., Lee J., Maynard H.D. (2012). Trehalose glycopolymers for stabilization of protein conjugates to environmental stressors. J. Am. Chem. Soc..

[26] Zhang B., Yao H., Qi H., Zhang X.-L. (2020). Trehalose and alginate oligosaccharides increase the stability of muscle proteins in frozen shrimp (*Litopenaeus vannamei*). Food Funct..

[27] Lesch A., Cortés-Salazar F., Amstutz V., Tacchini P., Girault H.H. (2015). Inkjet printed nanohydrogel coated carbon nanotubes electrodes for matrix independent sensing. Anal. Chem..

[28] Melnik E., Strasser F., Muellner P., Heer R., Mutinati G.C., Koppitsch G., Lieberzeit P., Laemmerhofer M., Hainberger R. (2016). Surface Modification of Integrated Optical MZI Sensor Arrays Using Inkjet Printing Technology. Procedia Eng..

[29] Bauer M., Duerkop A., Baeumner A.J. (2023). Critical review of polymer and hydrogel deposition methods for optical and electrochemical bioanalytical sensors correlated to the sensor’s applicability in real samples. Anal. Bioanal. Chem..

[30] Shafique H., de Vries J., Strauss J., Jahromi A.K., Moakhar R.S., Mahshid S. (2023). Advances in the Translation of Electrochemical Hydrogel-Based Sensors. Adv. Healthc. Mater..

[31] Tokuyama H., Nakahata Y., Ban T. (2020). Diffusion coefficient of solute in heterogeneous and macroporous hydrogels and its correlation with the effective crosslinking density. J. Membr. Sci..

[32] Johnson E.M., Berk D.A., Jain R.K., Deen W.M. (1996). Hindered diffusion in agarose gels: Test of effective medium model. Biophys. J..

[33] Zhou Y., Li J., Zhang Y., Dong D., Zhang E., Ji F., Qin Z., Yang J., Yao F. (2017). Establishment of a Physical Model for Solute Diffusion in Hydrogel: Understanding the Diffusion of Proteins in Poly(sulfobetaine methacrylate) Hydrogel. J. Phys. Chem. B.

[34] Axpe E., Chan D., Offeddu G.S., Chang Y., Merida D., Hernandez H.L., Appel E.A. (2019). A Multiscale Model for Solute Diffusion in Hydrogels. Macromolecules.

[35] Deffo G., Nde Tene T.F., Medonbou Dongmo L., Zambou Jiokeng S.L., Tonleu Temgoua R.C. (2024). Differential Pulse and Square-Wave Voltammetry as Sensitive Methods for Electroanalysis Applications.

[36] Caccavo D., Cascone S., Lamberti G., Barba A.A. (2018). Hydrogels: Experimental characterization and mathematical modelling of their mechanical and diffusive behaviour. Chem. Soc. Rev..

[37] Bray J.C., Merrill E.W. (1973). Poly(vinyl alcohol) hydrogels. Formation by electron beam irradiation of aqueous solutions and subsequent crystallization. J. Appl. Polym. Sci..

[38] Hickey A.S., Peppas N.A. (1995). Mesh size and diffusive characteristics of semicrystalline poly(vinyl alcohol) membranes prepared by freezing/thawing techniques. J. Membr. Sci..

[39] Lin S., Sangaj N., Razafiarison T., Zhang C., Varghese S. (2011). Influence of physical properties of biomaterials on cellular behavior. Pharm. Res..

[40] Selifonov A.A., Shapoval O.G., Mikerov A.N., Tuchin V.V. (2019). Determination of the Diffusion Coefficient of Methylene Blue Solutions in Dentin of a Human Tooth using Reflectance Spectroscopy and Their Antibacterial Activity during Laser Exposure. Opt. Spectrosc..

[41] Merrill E.W., Dennison K., Sung C. (1993). Partitioning and diffusion of solutes in hydrogels of poly(ethylene oxide). Biomaterials.

[42] Gil M.S., Cho J., Thambi T., Giang Phan V.H., Kwon I., Lee D.S. (2017). Bioengineered robust hybrid hydrogels enrich the stability and efficacy of biological drugs. J. Control. Release.

[43] Simon D., Obst F., Haefner S., Heroldt T., Peiter M., Simon F., Richter A., Voit B., Appelhans D. (2019). Hydrogel/enzyme dots as adaptable tool for non-compartmentalized multi-enzymatic reactions in microfluidic devices. React. Chem. Eng..

[44] Davari N., Bakhtiary N., Khajehmohammadi M., Sarkari S., Tolabi H., Ghorbani F., Ghalandari B. (2022). Protein-Based Hydrogels: Promising Materials for Tissue Engineering. Polymers.

