# Supplementary material for: Electrochemical Diffusion Study in Poly(Ethylene Glycol) Dimethacrylate-Based Hydrogels
Source: Sensors (Basel). 2024 Jun 6;24(11):3678. doi: 10.3390/s24113678 (PMC11175328; doi:10.3390/s24113678)
Supplement: Supplementary file 1 [file sensors-24-03678-s001.zip › sensors-2940683-supplementary.pdf]

SUPPORTING MATERIALS

Electrochemical Diffusion Study in Poly(Ethylene Glycol) Di-methacrylate-Based Hydrogels

Eva Melnik[1]*†, Steffen Kurzhals[1], Giorgio C. Mutinati[1], Valerio Beni[2] and Rainer Hainberger[1]

[1] Molecular Diagnostics, AIT Austrian Institute of Technology GmbH, 1210 Vienna, Austria;

[2] Bioelectronics and Organic Electronics, Smart Hardware, Digital Systems, RISE Research Institutes of Sweden, 60233 Norrköping, Sweden;

* Correspondence: eva.melnik@ait.ac.at

Content

2. Materials and Methods 2

2.2. Electrochemical Sensors and Measurement Setup 2

2.4. Gravimetric Hydrogel Characterization 3

2.5. Hydrogel Characterization with Scanning Electron Microscope 4

3. Results 5

3.2 Gravimetric Hydrogel Characterization 5

3.5. Response of MB-Conjugates of Sensors with and without Hydrogel 6

3.6 Hydrgelcharacteriazion with Different PEG-DMA Molecular Weights 6

2. Materials and Methods

2.2. Electrochemical Sensors and Measurement Setup

Figure S1 shows the measurement setup.

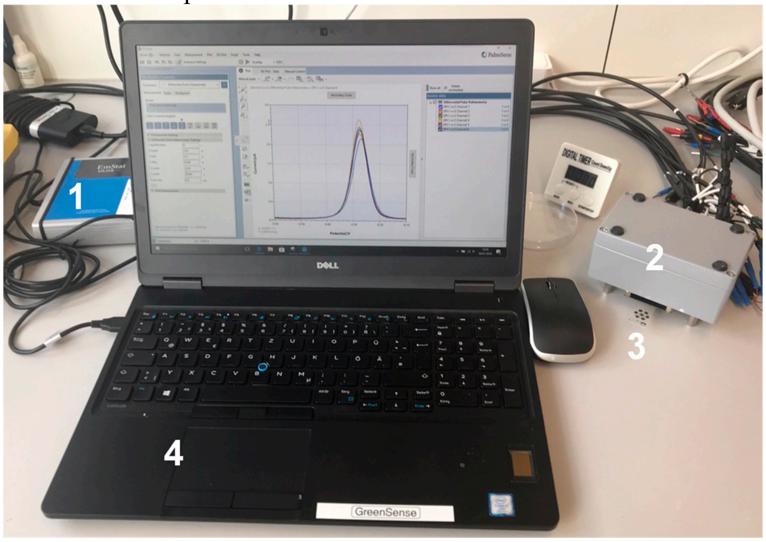

Figure S1: Measurement setup: 1) PalmSens multiplexer MUX8-R2 (PSTrace software 5.7), 2) connector, 3) sensor, 4) computer.

2.4. Gravimetric Hydrogel Characterization

Table S1: Variables of the gravimetric analysis

Name	Short	Formular	Determination
Number-average molecular weight of the polymer chain between the cross-links [1]	$\bar{M}_c$	$\frac{1}{\bar{M}_c} = \frac{2}{\bar{M}_n} - \frac{\tilde{v}/V_1[ln(1-v_{2,s}) + v_{2,s} + \chi v_{2,s}^2]}{v_{2,r}[\left(\frac{v_{2,s}}{v_{2,r}}\right)^{\frac{1}{3}} - \frac{v_{2,s}}{v_{2,r}}]}$	Calculation
Number-average of molecular weight	$\bar{M}_n$	Weight of the crosslinking polymer, PEG-DMA, 1-10kDa	From molecule specification
Specific volume of the polymer	$\tilde{v}$	$\tilde{v}=1/\rho_P$	With ρ_P, the bulk density of the polymer with 1.1 kg×L^{-1}
Molar volume of the solvent (18 cm^3/mol for water)	V_1	Molecular specification of water	18*10^-3 L×mol^{-1} for water
Flory-Huggin's polymer–solvent interaction parameter [1]	χ		0.495 for PEG-water system
Volume fraction of the relaxed gel	$v_{2,r}$	$v_{2,r}=V_P/V_r$	
Volume fraction of the swollen gel	$v_{2,s}$	$v_{2,s}=V_P/V_s$	
Relaxed volume of the polymer	V_r		Weighing and recording the weight after crosslinking (minus W_{sens}), and calculation of volume over the water density 1 mg/µl
Weight of the hydrogel after crosslinking	W_c		Weighing and recording the weight after crosslinking and subtraction of W_{sens}
Weight of the water in hydrogel	W_w	$W_w= W_{wash}- W_{dry}$	Calculation
Volume of the swollen gel	V_s		W_w in volume, 1 mg≙1 µl
Swollen hydrogel weight	W_{sh}	$W_{sh}= W_{wash}-W_{sens}$.	
Volume of the polymer	V_P	$V_P=W_P/\rho_P$	The density ρ_P is 1.1 kg×L^{-1}
Weight of the washed hydrogel	W_{wash}		Weighing and recording the weight of the sensor with hydrogel after initial swelling (washing)
Weight of the polymer	W_P	$W_p=W_{dry}-W_{sens}$	Calculation
Weight of the dried hydrogel after drying 24 h at room temperature	W_{dry}		Weighing and recording the weight of the sensor with hydrogel after initial swelling and drying
Weight of the screen-printed sensor	W_{sens}		Sensor weight, on which the hydrogel was fabricated
Mesh size [2]	ξ	$\xi = v_{2,s}^{-1/3} * C_n^{\frac{1}{2}} \left(\frac{2\bar{M}_c}{M_r}\right)^{\frac{1}{2}} * l$	Calculated
Rigidity factor of the polymer [3]	C_n		4 for PEG
Molecular weight of repeating units [2]	M_r		44 *10^-3 kg/mol for PEG
Carbon-carbon bond length [2]	l		0.154 nm
Swelling ratio	SR	$SR= W_{sh}/W_p$	

2.5. Hydrogel Characterization with Scanning Electron Microscope

To avoid electron charging effects, the hydrogel layers were fabricated on screen-printed silver layers for the imaging characterisation with the SEM. The pore structure of the hydrogel dried in a vacuum chamber was clearly visible, in contrast to hydrogels sputtered in a Cressington Sputter Coater 108auto before SEM analysis. The hydrogels were coated with a mixture of gold (Au) and palladium (Pd).

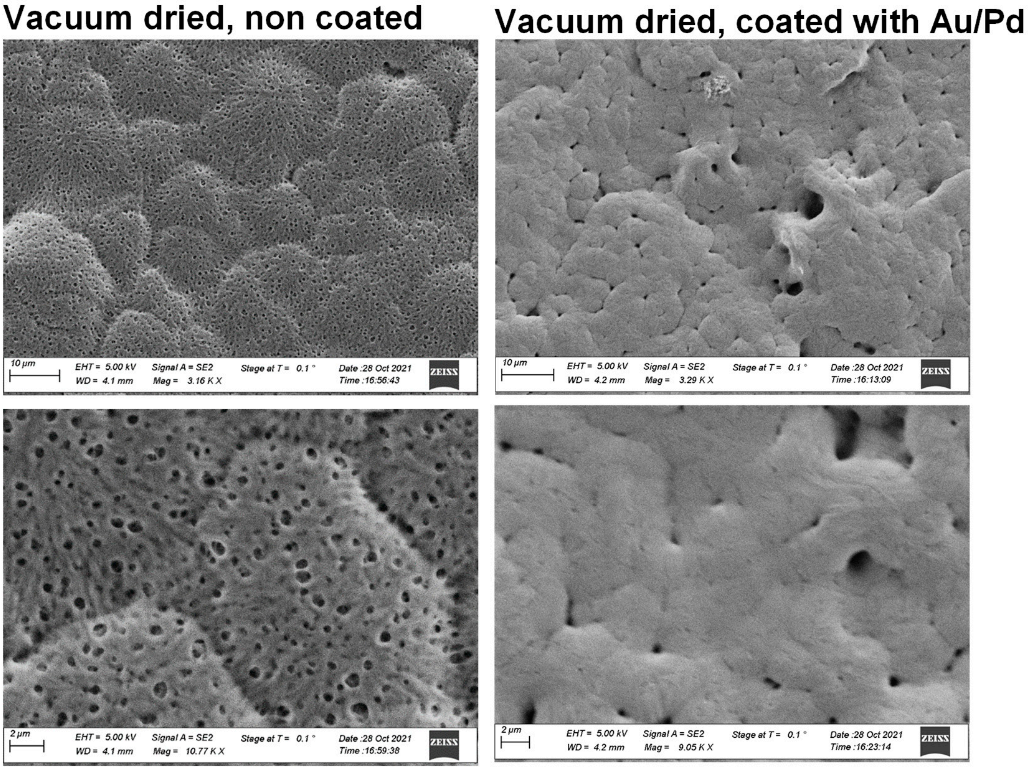

Figure S2: SEM images of vacuum dried and lyophilized hydrogel structures.

3. Results

3.2 Gravimetric Hydrogel Characterization

Table S2: Determined values form Gravimetric hydrogel characterization.

Molecular weight of PEG-DMA, Mw (kDa)	Molar concentration of PEG-DMA in the ink (MCI) (µmol/L)	PEG-DMA weight percent in the ink (%(w/w))	PEG-DMA Molar Concentration in the ink (µmol/L)	Weight of the polymer (W_p) (mg)	Swollen Hydrogel Weight (W_{sh}) (mg)	Swelling ratio $SR = W_{sh}/W_p$	Number-average molecular weight of the polymer chain between the cross-links, $\overline{M}_c$ (g/mol)	Mesh size ξ (nm)
**1**	54.1	5.4	54.1	7	101	14.43	407.37341	3.58
**2**	27.1	5.4	27.1	8.6	100.3	11.66	630.38039	4.46
**3.4**	15.9	5.4	16	14.7	140.4	9.53	900.60772	5.15
**10**	5.4	5.4	5.4	13.9	128.5	9.24	1238.2168	6.91

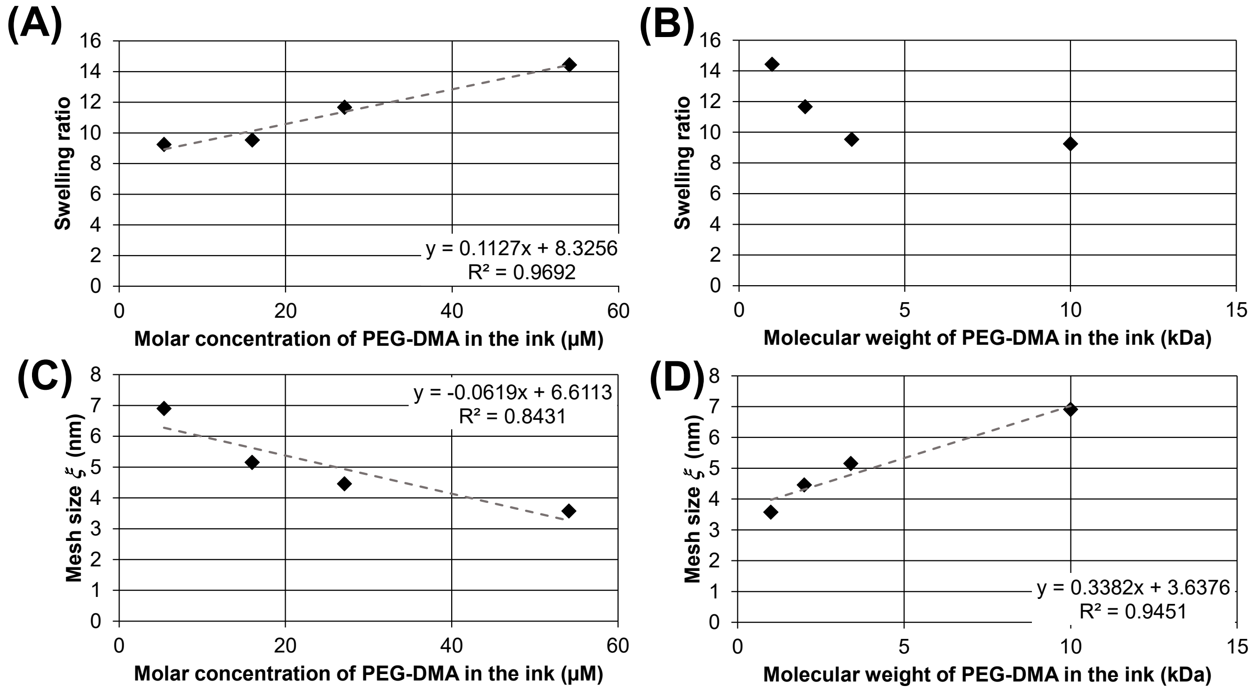

Figure S3: Correlation between (A) swelling ratio and the molar concentration in the ink, (B) swelling ratio and the molecular weight of PEG-DMA (kDa) (C) mesh size and the molar concentration in the ink (D) mesh size and the molecular weight of PEG-DMA (kDa).

3.5. Response of MB-Conjugates of Sensors with and without Hydrogel

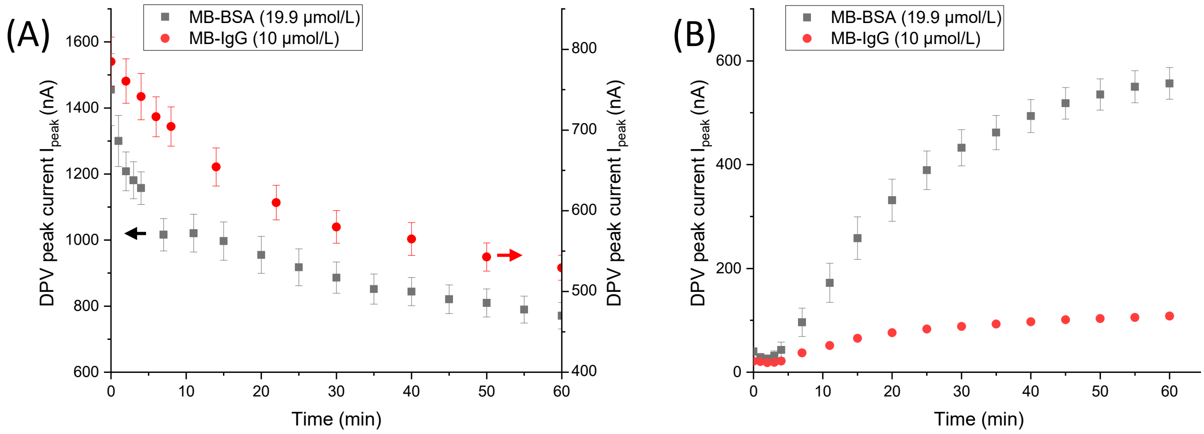

Figure S4: DPV measurement on electrochemical sensors of MB-BSA and MB-IgG (A) without and (B) with hydrogel (10kDa PEG-DMA as crosslinker) over 60min. During the first five minutes, a DPV measurement was performed every minute and afterwards every five minutes.

3.6 Hydrgel Characteriazion with Different PEG-DMA Molecular Weights

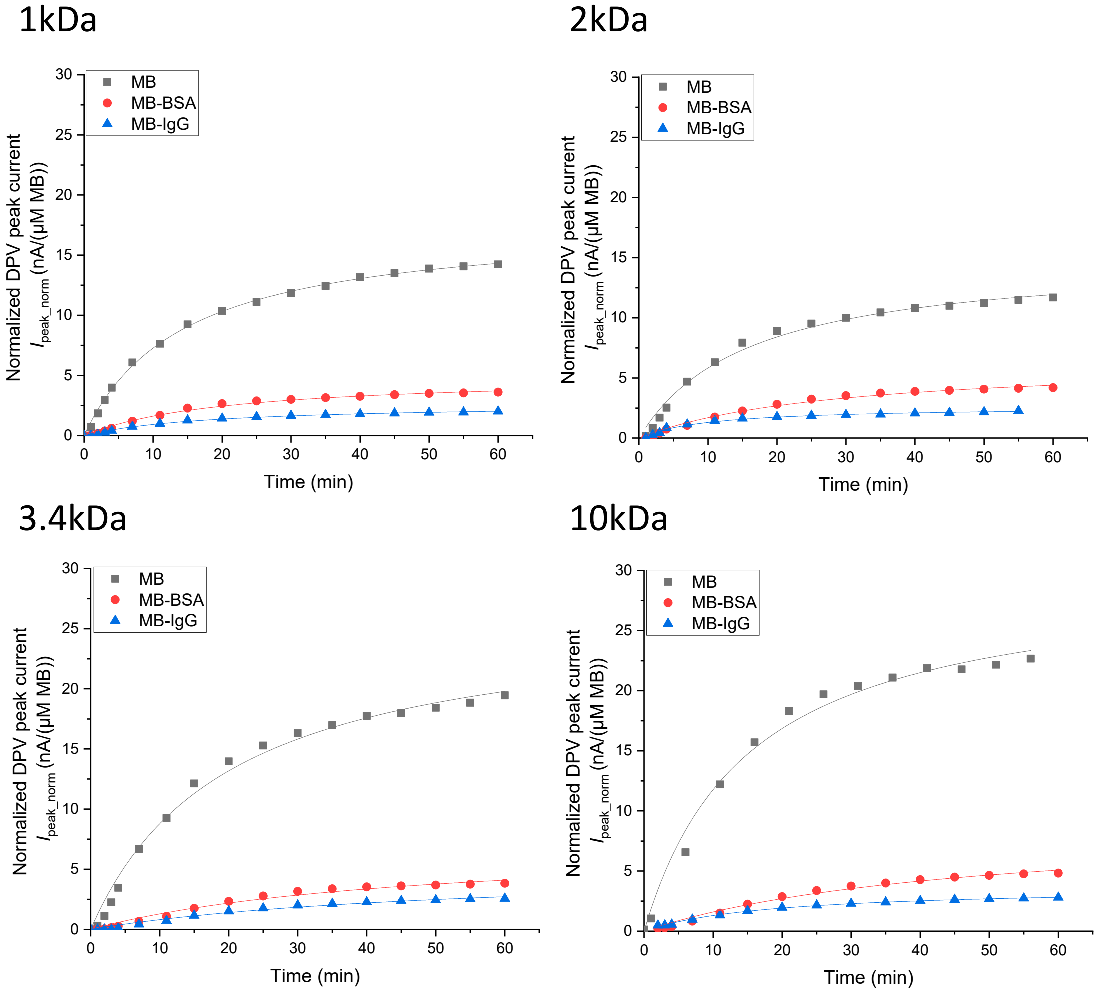

Figure S5: Normalized DPV peak currents (nA/µM MB) of MB, MB-BSA, and MB-IgG measurements on 1, 2, 3.4 and 10kDa PEG-DMA hydrogel modified sensors over 60 min. Curves were fitted with a Langmuir fitting function.

Table S3: Values of the equilibrium rate constant k_t.

PEG-DMA (kDa)	Methylene blue	MB-BSA	MB-IgG
	**k_t±sd, (min^{-1})**	**k_t±sd, (min^{-1})**	**k_t±sd, (min^{-1})**
**1**	14.4±0.5	21.1±2.5	18.8±1.9
**2**	15.9±1.7	28.1±3.7	10.3±1.2
**3.4**	20.1±2.1	46.5±10.6	53.8±2.5
**10**	15.2±1.9	46.5±8.0	18.4±1.0

1 Bray, J.C.; Merrill, E.W. Poly(vinyl alcohol) hydrogels. Formation by electron beam irradiation of aqueous solutions and subsequent crystallization. J. Appl. Polym. Sci. 1973, 17, 3779–3794, doi:10.1002/app.1973.070171219.
2 Hickey, A.S.; Peppas, N.A. Mesh size and diffusive characteristics of semicrystalline poly(vinyl alcohol) membranes prepared by freezing/thawing techniques. Journal of Membrane Science 1995, 107, 229–237, doi:10.1016/0376-7388(95)00119-0.

3 Lin, S.; Sangaj, N.; Razafiarison, T.; Zhang, C.; Varghese, S. Influence of physical properties of biomaterials on cellular behavior. Pharm. Res. 2011, 28, 1422–1430, doi:10.1007/s11095-011-0378-9.
